# 

**Published:** 1884-12-20

**Authors:** 


**To Our Subscribers.**—Please settle all back dues, and renew your subscriptions, promptly. See our liberal terms for renewal in our next item.

### EDITORIAL NOTICES.

OLEO CHYLE.—Examine the advertisement of this new preparation in the present number of our Journal.

An Electro-Therapeutic Association has been recently organized at Waco, Texas, and a similar Association is also proposed for the city of Austin.

Prof. Bemiss, an eminent man in the Profession in New Orleans, died recently at his residence in that city. Peace to his memory!

PRURITUS ANI.—The British Medical Journal says that Balsam of Peru is the best known remedy for pruritus ani.

Dr. F. E. Daniel, of Fort Worth, Texas, proposes to publish a Biography of Cotemporary Physicians of Texas.

CRAB ORCHARD WATER.—See the advertisement of this celebrated water in the present issue of our journal. It is said to be a liver regulator in addition to many other virtues.

IMPERIAL GRANUM.—This is, undoubtedly, an excellent food preparation for invaiids and aged people. For infants, also, it has been found a very wholesome and natural food. See the advertisement of this excellent article in this journal.

TOO MANY.—It is estimated that in Atlanta there is one *doctor* for every five hundred and fifty inhabitants. Take out the non-paying element from this number, and it gives poor encouragement to any who may contemplate coming to Atlanta to practice medicine.

### *RECEIPTED.*

1884.—Z. D. Emerson; F. C. Davis; F M. Fitzhugh; W. H. Johnson; A. J. Davis; J. C. Beauchamp: H. Perdue; C. C. Hammond; C. W. Kirby; A. R. Oglesby; J. S. Bushong; T. H. Lyon; J. F. Dorroh; J. T. Cleveland; D. C. Darnell; R. A. Mallory; J. R. Green; T. Courtney.

1883.—L. M. Wood; E. H. M. Parham; R. D. Simons; Edwin Smythe; J. Pepper; E. D. Logan.

